# Frequency of optimal and sub-optimal fertility-sparing surgery in young women with ovarian cancer: A single-center observational cohort study

**DOI:** 10.12669/pjms.42.(ICON26).15713

**Published:** 2026-04

**Authors:** Ayesha Saba, Muhammad Saqib Qamar Ishaqi, Faiza Rasheed, Shajeea Arshad Ali

**Affiliations:** 1 Dr. Ayesha Saba, FCPS Consultant Gynecologic Oncology, Department of Obstetrics and Gynecology, Indus Hospital and Health Network, Karachi, Pakistan; 2 Dr. Muhammad Saqib Qamar Ishaqi, FCPS Consultant, Department of Radiology, Indus Hospital and Health Network, Karachi, Pakistan; 3 Dr. Faiza Rasheed, FCPS Consultant, Department of Histopathology, Indus Hospital and Health Network, Karachi, Pakistan; 4 Dr. Shajeea Arshad Ali, MBBS Postgraduate Trainee, Department of Obstetrics and Gynecology, Indus Hospital and Health Network, Karachi, Pakistan

**Keywords:** CA-125, Computed tomography scans, Fertlity sparing surgery, Ovarian cancer

## Abstract

**Objective:**

Ovarian cancer (OC) is the leading cause of death among young women across the globe. The gold standard treatment is cytoreduction surgery, which is always challenging due to fertility desires. The objective of this study was to determine the frequency of fertility-sparing surgery (FSS) in young patients with OC.

**Methodology:**

A prospective observational cohort study was conducted at Indus Hospital and Health Network (IHHN), Karachi, Pakistan, from March 1, 2020, till June 30, 2024. This included women with diagnosed OC or epithelial borderline ovarian tumors (EBOT). The preoperative predictive scores of all women were evaluated, which indicated the best FSS outcomes.

**Results:**

A total of 15 patients with OC were recruited, out of which 14 (93.3%) had optimal FSS, while one (6.7%) patient had suboptimal FSS. Our patients were categorized as follows: five (33.33%) had germ-cell ovarian tumor, four (26.66%) had sex cord-stromal tumor, three had EBOT, and three had epithelial ovarian cancer. There was an association between tumor markers LDH and AFP with optimal FSS outcomes.

**Conclusions:**

Due to small sample size of our study, it is challenging to formulate a predictive model for optimal FSS. Hence, further prospective studies are required to design a predictive model for all types of OC.

## INTRODUCTION

Ovarian cancer (OC) is one of the fatal gynecological malignancies afflicting women across the globe.^1^ According to the International Agency for Research on Cancer (IARC), OC is the third most common female cancer in Pakistan, accounting for 4987 (5.1%) new cases reported in 2022. Consequently, the mortality rate from OC is also rising in Pakistan, accounting for 3492 (2.9%) deaths per year.^2^-^4^ This is attributed to a majority of women presenting in the advanced stages (III and IV) of cancer, while only 30% present in the early stages (I and II).^5^ The ideal treatment for OC is cytoreduction surgery with adjuvant chemotherapy.^6^ The treatment of OC in young women of reproductive age is always challenging due to the desire for fertility. The prevalence of OC in young women under 40 years of age is increasing, attributing to 13% of women presenting within this age bracket.^1^ The European Society of Gynecological Oncology (ESGO) guidelines proposed FSS in women with low grade 1-2 and stage 1A of EOC, including endometrioid, mucinous, low-grade serous and non-epithelial ovarian cancer, including malignant germ cell ovarian tumor (GCOC) and sex cord-stromal tumor (SCST).^7^-^8^

Several studies have reported favorable oncofertility outcomes in patients who underwent FSS due to OC.^9^-^10^ Based on International Ovarian Tumor Analysis (IOTA group), when the ovarian cyst shows malignant features, a PCT scan is recommended to rule out metastasis and consequently the advanced stage in which FSS is discouraged.^11^ Suidan et al. revealed the predictors for suboptimal debulking surgery using PCT scan findings such as massive ascites, liver parenchymal involvement, suprarenal lymphadenopathy, porta-hepatis involvement, intestinal involvement, extensive omental involvement, and diaphragmatic disease.^12^ Several studies have conferred that a preoperative serum cancer antigen 125 (CA-125) level of 420-500 IU/ml is one of the effective and independent predictors of suboptimal debulking surgery.^13^-^14^ Thus, it is imperative to formulate a predictive model using the aforementioned factors to select patients for FSS.

As per our knowledge, there is a dearth of prospective local studies on this topic; hence, this research will broaden the scope of the treatment modality of ovarian cancer in the current population. This study aimed to determine the frequency of optimal and suboptimal FSS along with frequencies of demographics, PCT scans and tumor markers in young patients with OC.

## METHODOLOGY

All women with diagnosed OC and EBOT who underwent FSS from March 1, 2020, till June 30, 2024, were recruited in the study. A prospective observational cohort study was conducted at IHHN, Karachi, Pakistan. All CT scans with standard contrast protocol were carried out within six weeks before surgery. Non-probability consecutive sampling technique was used.

### Ethical considerations:

This study was conducted after approval from the Institutional Ethical Review Committee (ERC) in 2020 with IRB reference number # (IHHN-IRB Number: IRD_IRB_2020_11_002/IHHN_IRB_2020_11_002; dated December 16, 2020). Informed consent was obtained from all participants.

### Inclusion criteria:

- All women aged 16-40 years with suspected or diagnosed OC or Epithelial Borderline Ovarian Tumor (EBOT) who underwent FSS at IHHN, Karachi, Pakistan, from March 1, 2020, till June 30, 2024.

### Exclusion criteria:

- Women with OC recurrence and women with OC who received neo-adjuvant chemotherapy were excluded from the study.

### Data Collection procedure:

Blood samples were taken within four weeks before surgery to measure tumor marker levels, including CA-125, lactate dehydrogenase (LDH), alpha-fetoprotein (AFP), and beta human chorionic gonadotrophin (B-HCG). A predictive scoring model was formulated using various variables to predict optimal FSS. A score of 0 to 2 was allotted to each parameter, with a combined score of 0 to 16 (Table-I). Five CT scan parameters were taken as the best predictors of optimal FSS based on Bristow scoring:

**Table-I:** Preoperative predictive scoring criteria.

	Predictors variables			Score 0		Score 1	Score 2
1	Age (years)			< 20		20-30	30-40
2	ECOG performance status			0		1	2 or more
3	Tumor marker levels	Epithelial ovarian cancer	CA-125 (IU/ml)	< 35	> 35 - < 500	> 500	―
		Germ cell ovarian tumors	AFP (ng/ml)	20	< 5000	> 5000	―
			BHCG (mIU/ml)	25	26 - < 500	> 500	―
			LDH (U/L)	< 225	> 225 - < 500	> 500	―
4	Volume of ascites			No		Mild-moderate (< 500 mL)	Massive (> 500 mL)
5	Peritoneal involvement			No		Thickening	Nodular
6	Omentum			No		Nodularity	Caking
7	Retroperitoneal lymph nodes			No		< l cm	> l cm
8	Diaphragm, lung bases or liver parenchyma metastasis			No		< 2cm	> 2cm

^a^ECOG: Eastern Cooperative Oncology Group; CA-125: Cancer antigen 125; AFP: Alpha-fetoprotein; B-HCG: Beta human chorionic gonadotrophin; LDH: lactate dehydrogenase. ^b^IU/mL: International units per milliliter; cm: centimeters; ng/mL: nanograms per milliliter; mIU/mL: milli-international units per milliliter; U/L: units per liter; mL: milliliter.

- Presence of ascites,
- Peritoneal thickening,
- Diaphragm, liver, and lung bases metastasis,
- Omentum involvement, and
- Pelvic and para-aortic lymph nodes.^14^

Besides CT scan parameters, the following new predictors were included in the predictive model: age, preoperative tumor marker levels, the American Society of Anesthesiologists (ASA) physical status classification system, and the Eastern Cooperative Oncology Group (ECOG) performance status (Table-I). FSS was performed by a trained gynecologic oncologist. Surgery comprised of a midline laparotomy including ascitic fluid cytology, unilateral salpingo-oophorectomy, biopsy of contralateral ovary, total omentectomy, pelvic and para-aortic lymphadenectomy, and resection of all visible tumor and appendectomy in mucinous ovarian cancer. The procedure aimed to keep residual disease 0 to < 1 cm.

### Statistical analysis:

All statistical analyses were performed using Statistical Package for the Social Sciences (SPSS version 24.0, IBM Corp., Armonk, NY, US). Descriptive Analysis was performed. Frequencies and percentages were reported for age, parity, marital status, BMI, comorbidities, ECOG performance score, FIGO stage, Histologic grade, histologic subtype, ASA class, optimal and suboptimal FSS.

## RESULTS

The study recruited 15 patients diagnosed with OC who underwent FSS in our tertiary care hospital. There were 14 (93.3%) patients who had optimal FSS, while only one (6.7%) patient had suboptimal FSS due to advanced stage. Most of our patients (n=8, 53.3%) were between 20-30 years. The majority of the patients, i.e., 14 (93.3%), were nulliparous. Eight (53.3%) patients had a BMI between 20-30 kg/m^2^, and four (26.6%) had a BMI greater than 30 kg/m^2^. Most of our patients (n=12, 73.3%) had no comorbidities, while two (13.3%) patients had controlled comorbidities. More than half of our patients (n=9, 60%) were classified as ASA class II, while five (33.3%) patients were graded as ASA class I. Similarly, 14 (93.3%) patients were categorized as having ECOG performance status 0, while one patient (6.7%) had ECOG performance status 1. Out of a total of 15 patients, five (33.3%) had GCOC, four (26.7%) had SCST, three (0.2%) had EBOT, and three (0.2%) had EOC. A majority of our patients (n=14, 93.3%) presented as stage 1 of OC as per the staging by the International Federation of Gynecology and Obstetrics (FIGO). Only one patient (6.7%) had suboptimal FSS due to advanced stage III, while 14 (93.3%) patients had optimal FSS (Table-II).

**Table-II:** Demographics and patient characteristics.

Demographics		Optimal FSS N (%)	Suboptimal FSS N (%)	Total number of cases N (%)
Age (years)	<20	2 (13.3%)	1 (6.7%)	3 (20%)
	20-30	8 (53.3%)	0	8 (53.3%)
	30-40	4 (26.6%)	0	4 (26.6%)
Parity	Nulliparous	13 (86.6%)	1 (6.7%)	14 (93.3%)
	1-5	1 (6.7%)	0	1 (6.7%)
Marital status	Single	7 (46.6%)	1 (6.7%)	8 (53.3%)
	Married	7 (46.6%)	0	7 (46.6%)
BMI (kg/m^2^)	<20	2 (13.3%)	1 (6.7%)	3 (20%)
	20-30	8 (53.3%)	0	8 (53.3%)
	>30	4 (26.6%)	0	4 (26.6%)
Comorbidities	None	11 (73.3%)	1 (6.7%)	12 (80%)
	Controlled	2 (13.3%)	0	2 (13.3%)
	Uncontrolled	1 (6.7%)	0	1 (6.7%)
ECOG performance status	0	13 (86.6%)	1 (6.7%)	14 (93.3%)
	1	1 (6.7%)	0	1 (6.7%)
	2	0	0	0
	3	0	0	0
FIGO Stage	I	14 (93.3%)	0	14 (93.3%)
	II	0	0	0
	III	0	1 (6.7%)	1 (6.7%)
	IV	0	0	0
Histological grade	I	7 (46.6%)	1 (6.7%)	8 (53.3%)
	II	6 (40%)	0	6 (40%)
	III	1 (6.7%)	0	1 (6.7%)
Histology subtype	Epithelial ovarian cancer	3 (20%)	0	3 (20%)
	Germ cell ovarian carcinoma	4 (26.6%)	1 (6.7%)	5 (33.3%)
	Sex-cord stromal tumor	4 (26.6%)	0	4 (26.6%)
	Epithelial borderline ovarian tumor	3 (20%)	0	3 (20%)
ASA class	I	5 (33.3%)	0	5 (33.3%)
	II	8 (53.3%)	1 (6.7%)	9 (60%)
	III	1 (6.7%)	0	1 (6.7%)
	IV	0	0	0

^a^FSS: Fertility-sparing surgery; ECOG: Eastern Cooperative Oncology Group performance status; BMI: Body mass index; FIGO: International Federation of Gynecology and Obstetrics; ASA class: American Society of Anesthesiologists physical status classification system; kg/m^2^: kilograms per square meter.

**Fig.1:**
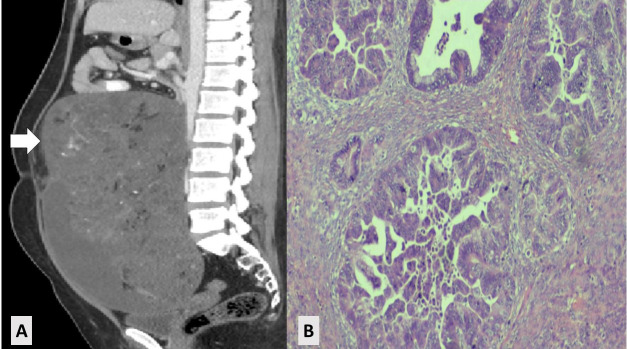
Mucinous ovarian carcinoma. A: Sagittal view of CT scan showing solid cystic pelvic lesion. B: Histopathology showing mucinous ovarian carcinoma. aCT scan: computed tomography scan.

The subtypes of histopathology of ovarian cancer are mentioned in Table-III. The common histological types found in our study were granulosa-cell ovarian tumors (n=3, 20%), dysgerminoma (n=3, 20%), mucinous borderline (n=3, 20%), and mucinous ovarian carcinoma (n=2, 13.3%). (Fig.1).

**Table-III:** Histopathology types of ovarian cancer (N=15).

S. No	Histological type	Number (n)	Percentage (%)
1	Granulosa cell ovarian tumor (Adult type)	3	20%
2	Dysgerminoma	3	20%
3	Endometrioid ovarian tumor	1	6.7%
4	Immature teratoma	1	6.7%
5	Mucinous borderline ovarian tumor	3	20%
6	Mucinous ovarian carcinoma	2	13.3%
7	Sertoli-Leydig cell tumor	1	6.7%
8	Yolk sac tumor	1	6.7%

**Fig.2:**
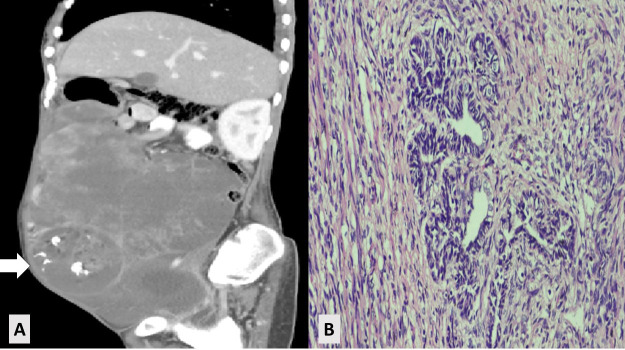
Immature teratoma. A: Sagittal view of CT scan showing multiloculated solid cystic pelvic lesion. B: Histopathology showing low-grade immature teratoma. aCT scan: computed tomography scan.

The less common types endometroid ovarian tumors, Sertoli-Leydig cell tumors, yolk cell tumors, and immature teratomas (Fig.2)

Five (33.3%) patients had CA-125 levels below 500 IU/mL and all underwent optimal FSS while only one (6.7%) patient had CA-125 levels above 500 IU/mL and underwent optimal FSS. Four (26.6%) patients had AFP levels below 5000 ng/mL and underwent optimal FSS while one (6.7%) patient had AFP levels above 5000 ng/mL and underwent optimal FSS. One (6.7%) out of two patients having LDH score below 500 U/L underwent optimal FSS while three (20%) patients having LDH levels above 500 U/L underwent optimal FSS (Table-IV).

**Table-IV:** Tumor marker levels and optimal and suboptimal FSS

	Tumor marker levels	Optimal FSS N (%)	Suboptimal FSS N (%)	Total number of cases N (%)
CA-125 (IU/mL)	< 500	5 (33.3%)	0	5 (33.3%)
	> 500	1 (6.7%)	0	1 (6.7%)
AFP (ng/mL)	< 5000	4 (26.6%)	0	4 (26.6%)
	> 5000	0	1 (6.7%)	1 (6.7%)
B-HCG (mIU/mL)	< 500	4 (26.6%)	1 (6.7%)	5 (33.3%)
	> 500	10 (66.6%)	0	10 (66.6%)
LDH (U/L)	< 500	1 (6.7%)	1 (6.7%)	2 (13.4%)
	> 500	3 (20%)	0	3 (20%)

^a^FSS: fertility-sparing surgery; CA-125: Cancer antigen 125; AFP: Alpha-fetoprotein; B-HCG: Beta human chorionic gonadotrophin; LDH: lactate dehydrogenase; IU/mL: International Units per milliliter; ng/mL: nanograms per milliliter; mIU/mL: milli-international units per milliliter; U/L: units per liter.

Out of a total of 14 patients who underwent optimal FSS, 10 (66.6%) had scores of 0-5, while four (26.6%) patients had scores of 6-8. Only one (6.7%) patient had a score greater than 9, who underwent suboptimal FSS. (Table-V).

**Table-V:** Predictive score and optimal and suboptimal FSS

Total predictive score	Optimal FSS, n (%)	Suboptimal FSS, n (%)	Total number of cases N (%)
Scores 0-5	10 (66.6%)	0	10 (66.6%)
Scores 6-8	4 (26.6%)	0	4 (26.6%)
Score ≥ 9	0	1 (6.7%)	1 (6.7%)

^a^FSS: fertility-sparing surgery.

## DISCUSSION

The management of OC in young women is based on both oncological and reproductive outcomes. This is vital in gynecologic oncology surgery, where FSS plays a massive role in uplifting the psychological and sexual wellness of a patient.^16^ This study aimed to assess the frequency of FSS and other variables in diagnosed OC or EBOT patients.

In our study, most patients (N=8, 53.3%) presented at ages ranging between 20-30 years, similar to another study by Chin F et al.^17^ The selection of patients for any treatment is based on a predictive model, including different prognostic factors as depicted by our study. Various studies supported that FSS can be performed up to stage 1.^7^, ^18^ In our study, 14 (93.3%) out of 15 patients underwent optimal FSS with FIGO 1 (Table-II). One study suggested the removal of both ovaries and preservation of the uterus and pregnancy via donor eggs in women with EBOT stage 1A grade 3, stage 1B and 1C, grade 1-2.^16^ Concurrently, SEER analysis found that the preservation of the uterus does not influence survival in stages 1A and 1C.^19^ Another past study showed promising fertility outcomes of FSS. In that study, out of 25 patients who underwent FSS, 15 women conceived, and 13 live births were recorded.^20^ A meta-analysis by Zhang et al. reported a recurrence rate of 12% in women who underwent FSS.^21^ So, it is vital to consider ovarian preservation while performing debulking surgery if the risks of spillage or recurrence are negligible.^22^

Our study found that 93.3% patients in the age range of 16-40 years and having either no or controlled/uncontrolled comorbidities underwent optimal FSS (Table-II). Comorbidities and age were not found to be significantly associated with optimal debulking surgery in a retrospective study conducted in Tehran (p-value >0.05).^13^

As per Song YJ et al., a preoperative CA-125 level > 500 U/mL is a significant predictor for suboptimal resection.^14^ Similarly, a study conducted in France by Depoers et al. proved that tumor markers were significantly associated with OC.^22^ Our study had Five (33.3%) patients with CA-125 levels below 500 IU/mL and all underwent optimal FSS while only one (6.7%) patient had CA-125 levels above 500 IU/mL and underwent optimal FSS. However, the increased levels of tumor markers, AFP and LDH were found to be significant predictive markers of optimal FSS in our study (Table-IV).

A total of 14 patients in our study had a total predictive score of < 9, and all underwent optimal FSS due to early stage of tumor (Table-V). Concomitantly, a Cochrane review concluded that FSS could be performed in stage 1 EBOT, stage 1A and 1C, grade 1 of EOC, and mixed germ cell tumor with good fertility outcomes and five-year overall survival rates.^16^

There is a dearth of literature on the subject of determinants for FSS in young women with OC using PCT scans and serum tumor markers. With 70% of ovarian cancers diagnosed at later stage, the known or identified prevalence remains low,^23^ hence, the sample size of this study also conforms with the current incidence rate globally accounting for 240,000 new cases, thereby which formulation of a predictive model for optimal FSS was not applicable. Therefore, further prospective multi-center studies with large sample size are desired to address this unique health issue.

## CONCLUSION

Due to the limited resources and small sample size of our study, it is challenging to formulate a predictive model for optimal FSS. Hence, further prospective studies are required to design an upgraded predictive model for all types of OC in young women.

### Authors’ contribution:

**AS: S**tudy concept and design, data analysis and manuscript writing and responsible for the accuracy of the study.

**MSQI FR:** Data collection & interpretation, critical review.

**SAA:** Literature search and manuscript writing.

All authors have approved the final version and are accountable for the integrity of the study.
